# Effect of COVID-19 Pandemic on Dietary Habits and Sleep Quality Applying the Pittsburgh Sleep Quality Index in Adult Saudi Population: A Cross-Sectional Study

**DOI:** 10.3390/ijerph191911925

**Published:** 2022-09-21

**Authors:** Hend F. Alharbi, Hassan Barakat

**Affiliations:** 1 Department of Food Science and Human Nutrition, College of Agriculture and Veterinary Medicine, Qassim University, Buraydah 51452, Saudi Arabia; 2 Food Technology Department, Faculty of Agriculture, Benha University, Moshtohor 13736, Egypt

**Keywords:** sleep quality, PSQI, dietary habits, COVID-19

## Abstract

The study aimed to evaluate the possible correlations between sleep quality and dietary habits in a population of Saudi during the COVID-19 pandemic. Exactly 444 adults completed a web-based cross-sectional study using an electronic questionnaire. Results indicate a significant difference between body mass index (BMI) and bad sleep quality. Smoking is linked to bad sleep quality. Both genders affected by coronavirus had a substantially bad quality compared to non-affected. An association between the degree of craving for sugar and bad sleep quality was found. In addition, there was a statistical difference between males and females who crave sugar very often in bad sleep quality. The result of sleep latency in males was 35.83%, who suffered from a severe sleep disorder, while 41.18% were female. The sleep duration was 65.00%, and 53.90% of males and females slept between 6 and 7 h per day. Sleep efficiency, measured according to the Pittsburgh questionnaire protocol, was measured in percentages, where a value of less than 65.00% is considered the lowest sleep efficiency. Females had a lower sleep efficiency of 25.49% compared to males (13.33%). These differences were statistically significant (*p* = 0.03). In conclusion, quality and sleep duration were impaired during the COVID-19 pandemic, and the observed changes were associated with diet.

## 1. Introduction

The COVID-19 pandemic impacts the physical health of infected people and the mental health and well-being of the general public [1]. Because of the restricted preventative measures, physical activity and sun exposure are decreased, both of which are necessary for optimal sleep. Additionally, it is not easy to engage in enjoyable activities due to social isolation. Indeed, sleep boosts the immune system to work properly, improves memory, stimulates tissue repair, lowers blood pressure, and helps cell regeneration. These alterations impair sleep and raise the risk of mental health disorders [2,3]. The pandemic affected sleep patterns and produced tension and anxiety [4,5,6]. Approximately 30% of American adults have sleeping disorders. A pre-pandemic study of Saudi people indicated that 33.80% sleep less than seven hours every night [7]. According to another survey, 41.50% of Saudi inhabitants are sleep deprived. A sufficient quantity of sleep prepares the body to withstand damage and illness, reduces the chance of infection, and boosts immunity against COVID-19. Difficult sleep deprivation deals with fatigue, high blood pressure, sleepiness, and a lowered immune system [8]. Epilepsy, depression, accidents, heart disease, diabetes, and a poor quality of life were linked to sleep disorders or insufficient sleep [9,10]. As a result, during the COVID-19 pandemic, numerous studies focused heavily on sleep and sleep disorders [1,11,12]. Huang and Zhao used the Pittsburgh sleep quality index (PSQI) to assess sleep quality in China (*n* = 603) and found that the Chinese population had poor sleep quality during the lockdown [13].

Furthermore, Yu et al. [14] found that most students reported that their sleep deteriorated due to the pandemic. Despite the evidence mentioned earlier, data on the COVID-19 lockdown and sleep disorders in the general population of Middle Eastern countries, particularly Saudi Arabia, are limited. A relationship between sleep and diet was hypothesized about 30 years ago in the context of behavioral factors affecting human health. Normal sleep duration is usually associated with higher caloric intake and absolute or relative intake of nutrients or food [15]. Among the potential mechanisms proposed to explain the effect of sleep on food intake are increased waking hours, increased frequency of meals (e.g., unhealthy snacks), altered mealtimes (e.g., late evening or night feeding) [16], and physiological effects of sleep deprivation on appetite (leptin and ghrelin) and metabolic (e.g., cortisol, insulin sensitivity and growth hormone) hormone homeostasis [17]. This hypothesis could also explain the relationship between sleep duration and high obesity rates [18]. In addition to its impact on metabolic disorders through an increased risk of obesity, several recent studies focused on sleep as a dietary supplement, showing an overall association between shorter sleep durations, lower dietary quality, and irregular eating behavior [19]. Thus, considering dietary intake, it is reported that people who sleep less than 1 h have a poor-quality diet [20]. To the best of our knowledge, this is the first study examining changes in dietary habits and sleep quality in Saudi adult residents during the COVID-19 pandemic. Therefore, a potential relationship between dietary habits and sleep quality in a Saudi population during the COVID-19 pandemic was investigated.

## 2. Materials and Methods

### 2.1. Study Design

A cross-sectional, internet-based questionnaire was performed according to the Helsinki Declaration principles and conducted among the Saudi population during COVID-19. The survey was carried out in Saudi Arabia over two months, from 1 February to 30 March 2022. Saudi citizens and non-Saudi residents who can read and understand the questionnaire were recruited using convenience sampling (Google Forms). We used WhatsApp and Twitter to reach our target population. Four hundred forty-four participants were recruited from different regions in Saudi Arabia.

### 2.2. Questionnaire

A self-administration questionnaire was used. Specialists examined the questionnaire in connected subjects. External reviewers also offered criticism and suggestions for creating/improving the questionnaire. The experts selected and self-connected based on experience and publishing similar studies in the human nutrition field. However, several changes were made to improve the questionnaire’s reliability, validity, and data collection’s scientific worth. Therefore, pilot research (*n* = 50 participants) was conducted to verify the validity and reliability of the questionnaire. Cronbach’s alpha was acquired and determined to be excellent, exceeding higher than 80% for measured parameters.

The survey comprises five sections: (1) the survey’s aim and online consent form, and (2) demographic information, including age, gender, nationality, education levels, occupation, marital status, income, and area of residence. (3) Health information, including body mass index (BMI) according to [21], smoking, and regular exercise. (4) How often you eat, how often you crave sweets, what you eat, what you eat most of, and how often you eat fast food are all factors in your diet (Likert-type scale [hereafter “Likert scale”]), and how much water you drink compared to how much you want coffee, tea, or soda (using the Likert scale). Dietary consumption was evaluated using a validated version of the Saudi Food and Drug Administration’s food frequency questionnaire (SFDA FFQ) a year before the study [22]. (5) An Arabic version of the Pittsburgh sleep quality index (PSQI) was employed [23]. Seven factors were assigned a score between 0 and 3: subjective sleep quality, sleep length, sleep latency, habitual sleep efficiency, usage of sleep medicines, sleep disruption, and daytime dysfunction. For instance, a score of 0 would indicate the absence of a sleep disorder, 1 would suggest a mild disruption, 2 would indicate a moderate disturbance, and a score of 3 would indicate a severe disturbance. Scores higher on the global PSQI (which range from 0 to 21) indicate a more serious sleep disturbance [24]. However, with a score below 5, sleep quality is poor.

### 2.3. Ethical Considerations

The study was conducted following the Declaration of Helsinki and approved by the Institutional Review Board (or Ethics Committee) of Qassim University No. 21-08-01 on 30 January 2022. Participants read the “Informed Consent” section at the beginning of the online form and checked the box indicating their agreement to the study’s terms before continuing. There is no identifying information in any of the data. In accordance with Google policy, all responses will be kept private and anonymous [25].

### 2.4. Statistical Analysis

Data were entered into Excel and analyzed with SPSS version 23.0. (IBM, Armonk, New York, NY, USA). The means and percentages were compared using an independent sample t-test and analysis of variance. For statistical significance, a *p*-value of less than 0.05 was used.

## 3. Results

### 3.1. Demographic Characteristics

In Table 1, four hundred forty-four participants responded to the survey; 95.9% were Saudi citizens. Of the respondents, 54.1% were males, and 45.9% were females. The response from the age group of 18 to 30 years was 40.90%, while 32.80% were between 31 and 39 years, 22.5% were in the age group of 41–59 years, and 3.6% were 60–69 years. Most participants were married, 95.90% and 39.60% did not have children, and 16.60% had 3–4 children. More than 56% of respondents have a bachelor’s degree, 15.70% obtained a high school degree or less, and around 19.30% were postgraduate or higher. Additionally, more than 33% worked in the government sector, while 14.80%, 9.40%, and 2.70% worked in the private, health, and freelance sectors, respectively. Students participating in this study were 16.22%, 5.40% were retired, and 17.50% did not work. Forty percent worked between 8 and 10 h per day, five percent worked more than 10 h per day, and thirty-six percent worked less than 8 h per day. A total of 28% of respondents were less than 5000 SR per month, salary incomes from 5001 to 9999 and 10,000 to 15,000 were equally around 25.20%. Most respondents were from the Makkah region (23.4%), 15.77% were from the Riyadh region, and 15.3% were from the Qassim region. More than 89% lived in cities, most of which owned villas (about 37.8%), followed by rent flats, 31.9%.

### 3.2. Health Information

The association between health and sleep quality is demonstrated in Table 2. There was a significant difference between BMI and bad sleep quality. Among the participants, 79.17% of men and 86.28% of women had the bad sleeping feature. Most participants were of normal weight (35.59%), followed by overweight (35.14%), whereas underweight participants demonstrated only (3.6%). The highest bad sleep quality was 36.67% for men with overweight status. For women, 39.22% were categorized with a bad sleeping index. Smoking men had bad sleep quality compared to females. Both genders affected by coronavirus had a substantially bad quality compared to non-affected. Furthermore, 4.95% had type 2 diabetes, 4.05% were asthma affected, 4.05% had obesity, 2.7% had hypertension, 3.6% had type 1 diabetes, and 0.45% had osteoporosis, cancer, and liver disease. For the physical activities during COVID-19, about 24.32, 36.04, and 16.67% of participants changed a lot, changed a bit, and not changed in their physical activity levels, respectively. In contrast, 22.97% of participants did not participate in the questionnaire.

### 3.3. Eating Habits, Eating Patterns, and Consumption

Table 3 presents the association between dietary habits and sleep quality results. The dietary habits changed significantly during COVID-19 and demonstrated statistical significance between good and bad sleep quality (*p* > 0.05), and diet changed more for the worse than changed for the better. Participants who did not follow a healthy dietary pattern statistically differed in significance between good and bad sleep quality in both genders. An association between the degree of craving for sugar and bad sleep quality was found. There was a statistical difference between males and females who crave sugar very often in bad sleep quality. Half of the respondents do not know what melatonin is, while 46.40% do not take melatonin, and 2.25% only take melatonin. In addition, 27.48% took multivitamins, 18.92% took vitamin D, and 14.41% took vitamin C. Additionally, there was a significant difference between taking the protein bar, multivitamin, vitamin D, and vitamin C in men and sleep quality. Men who consume energy bars and supplements are usually active and do more physical activity, which improves their sleeping patterns. Among all participants, more than 50% consumed about 1–1.5 L of water.

### 3.4. Sleep Quality

Most sleep quality factors described below show significant gender differences (Table 4). The component of sleep quality was 19.61% in females, while 13.3% in males was in a fairly bad category. Males suffered from a severe sleep disturbance at a rate of 35.83%, while females did so at a rate of 41.18% in the sleep latency component. Surprisingly, the sleep duration of less than 5 h for both genders was 0%, while 65.00% and 53.90% of males and females slept between 6 and 7 h per day. According to the Pittsburgh questionnaire methodology, sleep efficiency was quantified as a percentage, with values below 65% indicating poor sleep quality. Compared to males (13.33%), females had a lower rate of adequate sleep time (25.49%). However, significant differences were found between the two groups (*p* = 0.03). Females had severe sleep disturbances, about 6.86%, compared to males, 3.33%, with no statistical differences. There was no statistically significant difference between women and men who used sleep medication less than once a week. Females had a moderate sleep disorder in the daytime dysfunction component at 20.59% compared to men (20.0%). Finally, the quality of sleep of the population during the COVID-19 pandemic did not differ according to gender. It was poor quality for 86.27% of females compared to 79.17% of men; this difference was not statistically significant (Table 4).

## 4. Discussion

To the best of my knowledge, this is the first study to investigate the effect of the COVID-19 pandemic on dietary behaviors and sleep quality among Saudi residents. The present research depicted the changes in sleep quality and dietary habits after reducing restrictive measures. Findings show that about 82.40% of the participants had poor sleep quality. Moreover, female participants were associated with worse global PSQI, daytime dysfunction, sleep quality, distribution, and latency. The prevalence of overall poor sleep was higher in those participants affected by coronavirus compared to others, indicating that they have less prevalence with worse scores. To lower future risks of noncommunicable diseases, Saudi females must make an effort to increase physical activity, avoid sedentary behaviors, get enough sleep, and improve their eating practices [26]. However, AlMarzooqi [27] verified the correlation between healthy eating habits and elevated levels of physical activity and the link between poor eating habits, excessive sedentary behavior, and sleep deprivation among Saudi women. Evidently, Saudi female college students seem to have a lot of bad lifestyle habits. In 7236 self-selected Chinese volunteers, Huang and Zhao found a substantially lower prevalence of poor sleep quality (18.2%) [13]. They used a similar PSQI questionnaire in this study to assess sleep quality, but they used a higher cutoff point > 7, which resulted in an underestimating of the population’s sleep quality. Our study used a cutoff > 5 to distinguish between poor and good sleep quality. The PSQI questionnaire’s developers, Buysse et al. [24], tested this cutoff point > 5, and they found that it distinguished between good and poor sleepers with 89.6% sensitivity and 86.5% specificity. Huang and Zhao found no statistically significant relationship between participant age and sleep quality (*p* = 0.446). Interestingly, The frequency of poor sleep was higher in people who were overweight and obese. However, the data show no statistically significant correlation between participants’ PSQI scores and BMI [28]. Kong et al. [29] concluded that 8.6% (n = 442) of adults reported poor sleep quality in Ningbo. Intake of soy sauce and alcohol was positively associated with poor sleep quality, and consumption of dark fruits and water were positively associated with good sleep quality

Al-Musharaf [30] conducted a study in Saudi Arabia to evaluate the pandemic’s effects on healthy young Saudi women. The study provided data on various variables, including sleep quality, stress scale, anxiety, depression, and emotional eating scale. That study also stated that stress, anxiety, and depression negatively impacted everyone’s PSQI score but omitted to mention the average score or the prevalence of poor sleep quality. According to Alnofaiey et al. [31], 43.9% of Saudi physicians have sleep disorders, with physicians between the ages of 31 and 40 having a higher frequency.

This research shows that participants who were overweight and obese slept less than participants who were of normal weight and that more underweight and obese participants complained of poorer sleep during the COVID-19 pandemic than normal participants, suggesting a favorable profile of normal weight status for sleep. Obesity is linked to shorter sleep durations and more inferior sleep quality [32]. Despite being entirely inactive, sleep does not cause weight gain [33]. Short and lengthy sleep durations were linked to an increased risk of obesity. The risk of obesity was linked to both short and extended sleep durations [34].

Additionally, a shorter sleep time was linked to higher consumption of calories and fat [35]. Possible biological explanations for the link between shorter sleep duration and weight gain include hormone changes and metabolite production [36]. For instance, among overweight young people, adequate sleep duration was linked to reducing the urge for high-calorie foods [37]. Aldahash et al. [38] showed that BMI was greater among smokers and people who slept for shorter periods, but there was no correlation between BMI and the characteristics of other students. We advise taking steps to stop smoking and practicing in excellent sleeping habits. Healthy eating and physical activity, particularly among people with high BMI, are impacted by the COVID-19 pandemic [39,40]. Additionally, the lack of availability of fresh food due to lockdown increased consumption of highly processed foods and goods with long shelf life, which are typically heavy in salt, sugar, and saturated fat, and only temporarily give the feeling of being full [41].

### Strength and Limitation

In particular, this study aimed to examine the relationship between sleep and eating patterns in a Saudi community affected by the COVID-19 pandemic. This study’s findings demonstrate that sleep quality and duration were negatively impacted by the COVID-19 pandemic, with dietary changes being a contributing factor. Many caveats prevent this study from being broadly applicable. First, the study was conducted during a time of easing restrictions, so the sampling was not random. Results should be interpreted with caution due to the skewed gender ratio of the sample and the fact that the vast majority of the data were collected in Makkah, Riyadh, and Qassim in SA.

## 5. Conclusions

In conclusion, the COVID-19 epidemic influenced adults’ dietary habits and sleep patterns in Saudi Arabia. COVID-19 saw a marked shift in eating patterns, one that was statistically significant between excellent and bad sleep quality (*p* > 0.05) and one that was markedly worse than the previous dietary pattern. Overall, poor sleep quality affects 86.27% of women and 79.17% of men, although this difference is not statistically significant. Despite the study’s focus on the Saudi population, its findings may apply to the health system. They should encourage additional research in local contexts and policy decisions aimed at bettering women’s health. At this new phase in the slow return to normalcy, it is essential to emphasize positive improvements, such as a healthier diet, and encourage the purchase of food from local farmers. Those supermarkets and grocery stores close to people’s homes fulfill all biosecurity regulations. Resuming some physical activity and engaging in other activities that foster mental stability should be actively promoted.

## Figures and Tables

**Table 1:** Demographic characteristics.

Variables		All Participant (444)	Male (240 Cases) (%)	Female (204 Cases) (%)	*p*-Value
Age	18–23	62	16.67 ^abA^	10.78 ^bA^	0.57
	24–30	120	17.50 ^abB^	38.24 ^aA^	
	31–39	146	34.17 ^aA^	31.37 ^aA^	
	40–49	66	18.33 ^abA^	10.78 ^bA^	
	50–59	34	7.50 ^bA^	7.84 ^bA^	
	60–69	16	5.83 ^bA^	0.98 ^bA^	
Nationality	Saudi	426	96.67 ^aA^	95.10 ^aA^	0.011
	Non-Saudi	18	3.33 ^bA^	4.90 ^bA^	
Marital status	Single	182	40.00 ^aA^	42.16 ^abA^	0.059
	Married	230	57.50 ^aA^	45.10 ^aA^	
	Widower/divorce	32	2.50 ^bA^	12.75 ^bA^	
No. children	Non	176	35.00 ^aA^	45.10 ^aA^	0.060
	1 child	56	12.50 ^bA^	12.75 ^bcA^	
	2 children	86	15.83 ^bA^	23.53 ^bA^	
	3–4 Children	74	18.33 ^abA^	14.71 ^bcA^	
	5 and more children	52	18.33 ^abA^	3.92 ^cB^	
Occupation	Student	72	13.33 ^bA^	19.61 ^abA^	0.305
	Health sector	42	8.33 ^bA^	10.78 ^abA^	
	Government sector	150	43.33 ^aA^	22.55 ^abB^	
	Private sector	66	18.33 ^abA^	10.78 ^abA^	
	Freelancer	12	3.33 ^bA^	1.96 ^bA^	
	Retired	24	10.00 ^bA^	0.00 ^bA^	
	No work	78	3.33 ^bB^	34.31 ^aA^	
Education	High school or less	70	18.33 ^bA^	12.75 ^bA^	0.004
	Diploma	36	11.67 ^bcA^	3.92 ^bA^	
	Bachelor	252	56.67 ^aA^	56.86 ^aA^	
	Master	60	13.33 ^bcA^	13.73 ^bA^	
	Ph.D.	26	0.00 ^cB^	12.75 ^bA^	
Area of residence	Riyadh region	70	12.50 ^abB^	19.61 ^abA^	0.198
	Makkah region	104	19.17 ^aB^	28.43 ^aA^	
	Eastern region	26	6.67 ^abA^	4.90 ^bcA^	
	Medina region	20	3.33 ^abA^	5.88 ^bcA^	
	Qassim region	68	2.50 ^bB^	30.39 ^aA^	
	Tabuk region	26	10.83 ^abA^	0.00 ^cB^	
	Hail region	4	0.00 ^bA^	1.96 ^cA^	
	Northern Border region	30	10.83 ^abA^	1.96 ^cB^	
	Asir region	42	15.83 ^abA^	1.96 ^cB^	
	Jizan region	16	5.83 ^abA^	0.98 ^cA^	
	Najran region	6	1.67 ^bA^	0.98 ^cA^	
	Al Baha region	4	0.83 ^bA^	0.98 ^cA^	
	Al-Jawf region	28	10.00 ^A^	1.96 ^cB^	
Place of living	Urban	396	85.00 ^aA^	94.12 ^aA^	0.073
	Rural	48	15.00 ^aA^	5.88 ^aA^	
Incomes	Less than 5000 SR	126	19.17 ^aB^	39.22 ^aA^	0.201
	5000–9999 SR	112	28.33 ^aA^	21.57 ^abA^	
	10,000–15,000 SR	112	25.83 ^aA^	24.51 ^abA^	
	15,000–20,000 SR	54	15.83 ^aA^	7.84 ^bA^	
	Above 20,000 SR	40	10.83 ^aA^	6.86 ^bA^	
Type home of living	Rent flat	142	33.33 ^aA^	30.39 ^bA^	0.004
	Owned flat	76	19.17 ^bA^	14.71 ^cA^	
	Rent villa	34	5.83 ^cA^	9.80 ^cdA^	
	Owned villa	168	34.17 ^aB^	42.16 ^aA^	
	Private housing (military—employees etc.	24	7.50 ^cA^	2.94 ^dA^	
Is there a outplace	yes	250	52.50 ^aA^	60.78 ^aA^	0.355
	no	194	47.50 ^aA^	39.22 ^aA^	
Working hours per day	Less than 8 h	160	41.59 ^aA^	48.53 ^aA^	0.019
	From 8 to 10 h	178	51.33 ^aA^	45.59 ^aA^	
	More than 10 h	24	7.08 ^bA^	5.88 ^bA^	

^a,b,c,d^: No significant difference (*p* > 0.05) between any two means within the same column with the same superscripted letters. ^A,B^: No significant difference (*p* > 0.05) between any two means for the same attribute within the same row have the same superscript letter.

**Table 2:** The association between health information and sleep quality by gender.

Variables	Items	All Participants (444) (%)	Male (240 Cases)		Female (204 Cases)	
			Good Sleep Quality PSQI < 5	Bad Sleep Quality PSQI > 5	Good Sleep Quality PSQI < 5	Bad Sleep Quality PSQI > 5
BMI	Underweight	16 (3.60)	1.67 ^aA^	1.67 ^cA^	0.98 ^aA^	2.94 ^cA^
	Normal	158 (35.59)	7.50 ^aC^	20.83 ^bB^	4.90 ^aC^	39.22 ^aA^
	Overweight	156 (35.14)	5.00 ^aC^	36.67 ^aA^	5.88 ^aC^	21.57 ^bB^
	Obesity	114 (25.68)	6.67 ^aB^	20.00 ^bA^	1.96 ^aB^	22.55 ^bA^
Smoking	Yes	130 (29.28)	5.83 ^aB^	34.17 ^aA^	0.00 ^aB^	16.67 ^bAB^
	No	314 (70.72)	15.00 ^aB^	45.00 ^aA^	13.73 ^aB^	69.61 ^aA^
Coronavirus	Yes	160 (36.04)	5.83 ^aB^	30.83 ^bA^	4.90 ^aB^	30.39 ^bA^
	No	284 (63.96)	15.00 ^aB^	48.33 ^aA^	8.82 ^aB^	55.88 ^aA^
Chronic diseases	Heart disease	6 (1.35)	0.83 ^aB^	1.67 ^cdA^	0.00 ^bC^	0.00 ^dC^
	Dyslipidemia	6 (1.35)	0.00 ^aB^	2.50 ^bcA^	0.00 ^bB^	0.00 ^dB^
	DM1	16 (3.60)	0.00 ^aC^	3.33 ^bA^	0.98 ^abB^	2.94 ^abA^
	DM2	22 (4.95)	0.00 ^aC^	6.67 ^aA^	0.00 ^bC^	2.94 ^abB^
	Hypertension	12 (2.70)	0.83 ^aC^	1.67 ^cB^	0.00 ^bD^	2.94 ^abA^
	Obesity	18 (4.05)	0.83 ^aC^	3.33 ^bA^	1.96 ^aB^	1.96 ^bcB^
	Asthma	18 (4.05)	0.83 ^aB^	3.33 ^bA^	0.00 ^bC^	3.92 ^aA^
	Osteoporosis	2 (0.45)	0.00 ^aB^	0.00 ^eB^	0.00 ^bB^	0.98 ^cdA^
	Cancer	2 (0.45)	0.00 ^aB^	0.83 ^deA^	0.00 ^bB^	0.00 ^dB^
	Liver disease	2 (0.45)	0.00 ^aB^	0.00 ^eB^	0.00 ^bB^	0.98 ^cdA^
	Kidney disease	0 (0.00)	0.00 ^aA^	0.00 ^eA^	0.00 ^bA^	0.00 ^dA^
Physical activities during COVID-19	Changed a lot	108 (24.32)	1.67 ^aC^	25.82 ^aA^	1.96 ^aC^	18.63 ^bcB^
	Changed a bit	160 (36.04)	6.67 ^aB^	30.00 ^aA^	3.92 ^aB^	31.37 ^aA^
	Not changed	74 (16.67)	5.00 ^aB^	9.17 ^bAB^	5.88 ^aB^	13.73 ^cA^
	I do not practice	102 (22.97)	7.50 ^aC^	14.17 ^bB^	1.96 ^aC^	22.55 ^bA^

^a,b,c,d,e^: No significant difference (*p* > 0.05) between any two means within the same column with the same superscripted letters. ^A,B,C,D^: No significant difference (*p* > 0.05) between any two means for the same attribute within the same row have the same superscripted letters.

**Table 3:** Eating habits, eating patterns, and consumption in the study population by gender are associated with sleep quality.

Variables	Items	All Participants (444) (%)	Male (240 Cases)		Female (204 Cases)	
			Good Sleep Quality PSQI < 5	Bad Sleep Quality PSQI > 5	Good Sleep Quality PSQI < 5	Bad Sleep Quality PSQI > 5
Dietary habits during COVID-19	Change a lot for the better	38 (8.56)	0.00 ^cB^	8.33 ^cA^	0.98 ^bB^	7.84 ^cA^
	Change a little for the better	134 (30.18)	5.83 ^bC^	23.33 ^aB^	3.92 ^abC^	27.45 ^aA^
	Not changed	156 (35.14)	12.50 ^aB^	25.00 ^aA^	7.84 ^aC^	24.51 ^aA^
	A much change for the worse	66 (14.86)	2.50 ^bcB^	13.33 ^bA^	0.98 ^bB^	12.75 ^bA^
	Little change for the worse	50 (11.26)	0.00 ^cC^	9.17 ^bcB^	0.00 ^bC^	13.73 ^bA^
Healthy dietary patterns	Yes	72 (16.22)	2.50 ^aB^	9.17 ^cAB^	4.90 ^aB^	16.67 ^bA^
	No	216 (48.65)	15.00 ^aC^	45.00 ^aA^	3.92 ^aD^	31.37 ^aB^
	Sometimes	156 (35.14)	3.33 ^aB^	25.00 ^bA^	4.90 ^aB^	38.24 ^aA^
No. meal/day	One meal	22 (4.95)	0.00 ^aA^	3.33 ^bA^	0.98 ^aA^	5.88 ^bA^
	Two meals	248 (55.86)	9.17 ^aB^	42.50 ^aA^	6.86 ^aB^	53.92 ^aA^
	Three meals	142 (31.98)	10.00 ^aBC^	28.33 ^aA^	4.90 ^aC^	19.61 ^bAB^
	More than three meals	32 (7.21)	1.67 ^aA^	5.00 ^bA^	0.98 ^aA^	6.86 ^bA^
Primary meal/day	Breakfast	100 (22.52)	4.17 ^aB^	10.00 ^bB^	2.94 ^aB^	29.41 ^aA^
	Lunch	210 (47.30)	9.17 ^aB^	36.67 ^aA^	7.84 ^aB^	41.18 ^aA^
	Dinner	134 (30.18)	7.50 ^aBC^	32.50 ^aA^	2.94 ^aC^	15.69 ^bB^
Degree of craving for sugar	Very often	104 (23.42)	1.67 ^aC^	15.00 ^bB^	0.98 ^aC^	30.39 ^aA^
	Often	86 (19.37)	2.50 ^aB^	13.33 ^bcA^	4.90 ^aB^	18.63 ^bA^
	Sometimes	168 (37.84)	8.33 ^aB^	31.67 ^aA^	6.86 ^aB^	28.43 ^aA^
	Almost never	70 (15.77)	7.50 ^aB^	14.17 ^bcA^	0.98 ^aC^	7.84 ^cB^
	Never	16 (3.60)	0.83 ^aA^	5.00 ^cA^	0.00 ^aA^	0.98 ^cA^
Degree of craving for tea	Very often	112 (25.23)	6.67 ^aB^	20.83 ^aA^	0.98 ^aC^	21.57 ^abA^
	Often	92 (20.72)	4.17 ^aC^	20.00 ^aA^	0.98 ^aC^	15.69 ^bB^
	Sometimes	138 (31.08)	5.83 ^aB^	27.50 ^aA^	2.94 ^aB^	25.49 ^aA^
	Almost never	78 (17.57)	4.17 ^aB^	8.33 ^bB^	5.88 ^aB^	17.65 ^bA^
	Never	24 (5.41)	0.00 ^aB^	2.50 ^bAB^	2.94 ^aAB^	5.88 ^cA^
Degree of craving for coffee	Very often	192 (43.24)	3.33 ^aC^	30.00 ^aB^	6.86 ^aC^	48.04 ^aA^
	Often	78 (17.57)	4.17 ^aBC^	18.33 ^abA^	0.98 ^aC^	10.78 ^bB^
	Sometimes	116 (26.13)	10.00 ^aBC^	20.83 ^aA^	4.90 ^aC^	15.69 ^bAB^
	Almost never	38 (8.56)	0.83 ^aA^	7.50 ^bcA^	0.98 ^aA^	7.84 ^bA^
	Never	20 (4.50)	2.50 ^aA^	2.50 ^cA^	0.00 ^aA^	3.92 ^bA^
Black coffee consumption	Once a day	148 (33.33)	4.17 ^abC^	22.50 ^aB^	1.96 ^aC^	39.22 ^aA^
	2–3 per day	60 (13.51)	0.83 ^bC^	11.67 ^bA^	5.88 ^aB^	8.82 ^bAB^
	4–5 times a day	12 (2.70)	0.00 ^bB^	4.17 ^bcA^	0.00 ^aB^	0.98 ^bAB^
	More than 5 times a day	6 (1.35)	0.00 ^bA^	2.50 ^cA^	0.00 ^aA^	0.00 ^bA^
	Once a week	28 (6.31)	1.67 ^bB^	8.33 ^bcA^	0.00 ^aB^	1.96 ^bB^
	2–4 per week	24 (5.41)	1.67 ^bB^	1.67 ^cB^	0.98 ^aB^	6.86 ^bA^
	5–6 per week	2 (0.045)	0.00 ^bA^	0.00 ^cA^	0.00 ^aA^	0.98 ^bA^
	1–3 per month	18 (4.05)	0.83 ^bA^	4.17 ^bcA^	0.98 ^aA^	1.96 ^bA^
	Less than once per month/never	146 (23.88)	11.67 ^aB^	24.17 ^aA^	3.92 ^aC^	25.49 ^aA^
Arabic coffee consumption	Once a day	164 (36.94)	8.33 ^aC^	25.00 ^aB^	4.90 ^aD^	36.27 ^aA^
	2–3 per day	66 (14.86)	2.50 ^abC^	15.00 ^bA^	2.94 ^aC^	8.82 ^bcB^
	4–5 times a day	20 (4.50)	0.83 ^bAB^	4.17 ^deA^	0.00 ^aB^	3.92 ^cdAB^
	More than 5 times a day	12 (2.70)	1.67 ^bA^	2.50 ^deA^	0.98 ^aA^	0.00 ^dA^
	Once a week	64 (14.41)	2.50 ^abB^	11.67 ^bcA^	2.94 ^aB^	11.76 ^bA^
	2–4 per week	42 (9.26)	0.83 ^bC^	6.67 ^cdeB^	0.98 ^aC^	10.78 ^bA^
	5–6 per week	6 (1.35)	0.00 ^bA^	0.83 ^eA^	0.00 ^aA^	1.96 ^dA^
	1–3 per month	28 (6.31)	1.67 ^bB^	5.83 ^deA^	0.98 ^aB^	3.92 ^cdAB^
	Less than once per month/never	42 (9.46)	2.50 ^abB^	7.50 ^cdA^	0.00 ^aB^	8.82 ^bcA^
Red/black tea consumption	Once a day	158 (35.59)	8.33 ^aC^	23.33 ^aB^	4.90 ^aC^	35.29 ^aA^
	2–3 per day	100 (22.52)	4.17 ^abC^	22.50 ^aA^	0.98 ^aD^	16.67 ^bB^
	4–5 times a day	30 (6.76)	2.50 ^abB^	8.33 ^bA^	0.00 ^aB^	1.96 ^dB^
	More than 5 times a day	16 (3.60)	1.67 ^abAB^	4.17 ^bA^	0.00 ^aB^	0.98 ^dAB^
	once a week	36 (8.11)	1.67 ^abB^	5.83 ^bA^	2.94 ^aAB^	5.88 ^cdA^
	2–4 per week	20 (4.50)	0.83 ^bB^	3.33 ^bAB^	0.00 ^aB^	4.90 ^dA^
	5–6 per week	18 (4.05)	0.83 ^bB^	5.83 ^bA^	0.00 ^aB^	0.98 ^dB^
	1–3 per month	20 (4.50)	0.00 ^bB^	1.67 ^bB^	0.98 ^aB^	6.86 ^cdA^
	Less than once per month/never	46 (10.36)	0.83 ^bB^	4.17 ^bB^	3.92 ^aB^	12.75 ^bcA^
Green tea consumption	Once a day	82 (18.47)	4.17 ^abB^	13.33 ^bA^	1.96 ^aB^	17.65 ^bA^
	2–3 per day	10 (2.25)	0.00 ^bA^	1.67 ^cA^	0.00 ^aA^	2.94 ^cA^
	4–5 times a day	4 (0.90)	0.00 ^bA^	1.67 ^cA^	0.00 ^aA^	0.00 ^dA^
	More than 5 times a day	2 (0.45)	0.00 ^bA^	0.83 ^cA^	0.00 ^aA^	0.00 ^dA^
	Once a week	48 (10.81)	0.00 ^bB^	7.50 ^bcA^	2.94 ^aB^	11.76 ^bcA^
	2–4 per week	26 (5.86)	0.83 ^bB^	4.17 ^bcAB^	0.00 ^aB^	6.86 ^cdA^
	5–6 per week	10 (2.25)	1.67 ^bA^	0.83 ^cA^	0.98 ^aA^	0.98 ^dA^
	1–3 per month	28 (6.31)	2.50 ^abA^	3.33 ^cA^	1.96 ^aA^	4.90 ^cdA^
	Less than once per month/never	234 (52.70)	11.67 ^aC^	45.83 ^aA^	5.88 ^aD^	41.18 ^aB^
Fast food consumption	Once a day	80 (18.02)	2.50 ^abB^	16.67 ^abA^	1.96 ^aB^	14.71 ^bA^
	2–3 per day	26 (5.86)	0.83 ^abC^	6.67 ^cdA^	0.00 ^aC^	3.92 ^cB^
	4–5 times a day	6 (1.35)	0.83 ^abA^	1.67 ^dA^	0.00 ^aA^	0.00 ^cA^
	More than 5 times a day	4 (0.90)	0.00 ^bA^	1.67 ^dA^	0.00 ^aA^	0.00 ^cA^
	Once a week	108 (24.32)	3.33 ^abC^	18.33 ^aB^	4.90 ^aC^	22.55 ^aA^
	2–4 per week	70 (15.77)	4.17 ^abC^	10.00 ^cB^	2.94 ^aC^	14.71 ^bA^
	5–6 per week	12 (2.70)	0.83 ^abAB^	1.67 ^dAB^	0.00 ^aB^	2.94 ^cA^
	1–3 per month	60 (13.51)	2.50 ^abB^	11.67 ^bcA^	0.98 ^aB^	11.76 ^bA^
	Less than once per month/never	78 (17.57)	5.83 ^aC^	10.83 ^cB^	2.94 ^aD^	15.69 ^bA^
Soda consumption	Once a day	98 (22.07)	5.83 ^aB^	17.50 ^aA^	1.96 ^abC^	18.63 ^bA^
	2–3 per day	38 (8.56)	2.50 ^abBC^	10.00 ^bcA^	0.00 ^bC^	3.92 ^dB^
	4–5 times a day	0 (0)	0.00 ^bA^	0.00 ^eA^	0.00 ^bA^	0.00 ^dA^
	More than 5 times a day	6 (1.35)	0.00 ^bA^	2.50 ^eA^	0.00 ^bA^	0.00 ^dA^
	Once a week	70 (15.77)	2.50 ^abB^	14.17 ^abA^	0.98 ^bB^	13.73 ^bcA^
	2–4 per week	56 (12.61)	4.17 ^abC^	8.33 ^cdB^	0.98 ^bD^	11.76 ^cA^
	5–6 per week	16 (3.60)	0.83 ^abB^	4.17 ^deA^	0.00 ^bB^	1.96 ^dB^
	1–3 per month	46 (10.36)	1.67 ^abC^	5.00 ^cdeB^	2.94 ^abC^	11.76 ^cA^
	Less than once per month/never	114 (25.68)	3.33 ^abD^	17.50 ^aB^	6.86 ^aC^	24.51 ^aA^
Melatonin intake	Yes	10 (2.25)	0.00 ^aA^	1.67 ^bA^	0.00 ^aA^	2.94 ^bA^
	No	206 (46.40)	7.50 ^aB^	35.83 ^aA^	8.82 ^aB^	41.18 ^aA^
	Do not know what melatonin is	228 (51.35)	13.33 ^aB^	41.67 ^aA^	4.90 ^aB^	42.16 ^aA^
Supplement	Protein bars or supplements	26 (5.86)	0.00 ^aB^	6.67 ^abA^	1.96 ^aB^	2.94 ^dAB^
	Multivitamin	122 (27.48)	0.83 ^aD^	14.17 ^aB^	5.88 ^aC^	36.27 ^aA^
	Vitamin D	84 (18.92)	0.00 ^aD^	6.67 ^abB^	4.90 ^aC^	28.43 ^aA^
	Vitamin C	64 (14.41)	0.00 ^aC^	8.33 ^abB^	3.92 ^aC^	17.65 ^bA^
	Vitamin B	40 (9.01)	0.00 ^aB^	2.50 ^bB^	3.92 ^aB^	12.75 ^bcA^
	Folic	26 (5.86)	0.00 ^aB^	0.83 ^bB^	0.98 ^aB^	10.78 ^bcA^
	Iron	44 (9.91)	0.00 ^aB^	2.50 ^bB^	2.94 ^aB^	15.69 ^bcA^
	Omega 3	32 (7.21)	0.83 ^aB^	4.17 ^bB^	0.98 ^aB^	8.82 ^cdA^
Water consumption	Less than 500 ml	108 (24.32)	6.67 ^aBC^	11.67 ^bB^	3.92 ^aC^	27.45 ^bA^
	1–1.5 L	224 (50.45)	10.83 ^aB^	40.83 ^aA^	7.84 ^aB^	41.18 ^aA^
	1.6–2 L	72 (16.22)	1.67 ^aB^	17.50 ^bA^	1.96 ^aB^	10.78 ^cA^
	More than 2 L	40 (9.01)	1.67 ^aB^	9.17 ^bA^	0.00 ^aB^	6.86 ^cAB^

^a,b,c,d,e^: No significant difference (*p* > 0.05) between any two means within the same column with the same superscripted letters. ^A,B,C,D^: No significant difference (*p* > 0.05) between any two means for the same attribute within the same row have the same superscripted letters.

**Table 4:** Sleep quality in the study population by gender.

Variables	Items	Male (240 Cases)		Female (204 Cases)		*p*-Value
		No.	%	No.	%	
Sleep quality	Very good	80	33.33 ^abA^	78	38.24 ^aA^	
	Fairly good	108	45.00 ^aA^	64	31.37 ^aA^	0.049
	Fairly bad	32	13.33 ^bcA^	40	19.61 ^abA^	
	Very bad	20	8.33 ^cA^	22	10.78 ^bA^	
Sleep latency	No sleep disorder	0	0.00 ^bA^	2	0.98 ^cA^	
	Mild sleep disorder	82	34.17 ^aA^	60	29.41 ^bA^	0.003
	Moderate sleep disorder	72	30.00 ^aA^	58	28.43 ^bA^	
	Severe sleep disorder	86	35.83 ^aA^	84	41.18 ^aA^	
Sleep duration	>7 h	22	9.17 ^bcA^	42	20.59 ^bcA^	
	6–7 h	156	65.00 ^aA^	110	53.92 ^aA^	0.010
	5–6 h	62	25.83 ^bA^	52	25.49 ^bA^	
	<5 h	0	0.00 ^cA^	0	0.00 ^cA^	
Habitual sleep efficiency	>85%	140	58.33 ^aA^	94	46.08 ^aA^	
	75–84%	48	20.00 ^bA^	34	16.67 ^bA^	0.032
	65–74%	20	8.33 ^bA^	24	11.76 ^bA^	
	<65%	32	13.33 ^bA^	52	25.49 ^abA^	
Sleep disturbances	No sleep disorder	22	9.17 ^bA^	4	1.96 ^bA^	
	Mild sleep disturbance	156	65.00 ^aA^	88	43.140 ^aA^	0.092
	Moderate sleep disturbance	54	22.50 ^abA^	98	48.04 ^aA^	
	Severe sleep disturbance	8	3.33 ^bA^	14	6.86 ^abA^	
Use of sleep medication	Not during the past month	80	33.33 ^abA^	78	38.24 ^aA^	
	Less than once a week	108	45.00 ^aA^	64	31.37 ^abA^	0.049
	Once or twice a week	32	13.33 ^bcA^	40	19.61 ^abA^	
	Three or more times a week	20	8.33 ^cA^	22	10.78 ^bA^	
Daytime dysfunction	No sleep disorder	110	45.83 ^aA^	64	31.37 ^abA^	
	Mild sleep disorder	68	28.33 ^abA^	78	38.24 ^aA^	0.071
	Moderate sleep disorder	48	20.00 ^bA^	42	20.59 ^abA^	
	Severe sleep disorder	14	5.83 ^bA^	20	9.80 ^bA^	
Global sleep score	Good sleep quality	50	20.83 ^aA^	28	13.73 ^aA^	
	Poor sleep quality	190	79.17 ^aA^	176	86.27 ^aA^	0.069

^a,b,c^: No significant difference (*p* > 0.05) between any two means within the same column with the same superscripted letters. ^A^: No significant difference (*p* > 0.05) between any two means for the same attribute within the same row have the same superscripted letters.

## Data Availability

Data is contained within the article.
